# Effectiveness of manual dexterity assessment methods for preclinical training in Dentistry

**DOI:** 10.1371/journal.pone.0311973

**Published:** 2024-12-04

**Authors:** Luis Eduardo Genaro, Tamíris da Costa Neves, Júlia Margato Pazos, Lívia Nordi Dovigo, Patrícia P. N. S. Garcia

**Affiliations:** 1 Postgraduate Program in Collective Health in Dentistry, School of Dentistry, São Paulo State University (UNESP), Araçatuba, São Paulo, Brazil; 2 Department of Social Dentistry, School of Dentistry, São Paulo State University (UNESP), Araraquara, São Paulo, Brazil; Transilvania University of Brasov: Universitatea Transilvania din Brasov, ROMANIA

## Abstract

**Objective:**

This study aimed to verify the predictive capacity of manual dexterity assessment methods for pre-clinical training in Dentistry.

**Method:**

Students from the fifth year of the undergraduate course in Dentistry (N = 95) participated in this study. Manual dexterity was investigated as a variable of interest, measured by the O’Connor Finger Dexterity Test methods—Model 32021, Purdue Pegboard Test—Model 32020A, Dental Manual Dexterity Assessment—DMDA, Class One Cavity Preparation Assessent—COCA and Class One Composite Resin Restoration Assessment—COCRA. The average score obtained in the evaluation of the quality of the cavity preparations and restorations was considered as gold standard, and from these data the sensitivity and specificity of the tests were estimated. Receiver Operating Characteristic (ROC) curves were constructed to assess the diagnostic performance of each dexterity test. The analysis included calculating the Area Under the Curve (AUROC) to evaluate their discriminative power, and cutoff points were determined that optimize the balance between sensitivity and specificity.

**Results:**

The DMDA test showed better performance, with statistical significance (p <0.001) and acceptable predictive ability (AUROC = 0.775), while the O’Connor test (AUROC = 0.644, p = 0.050) and Purdue Pegboard test (Purdue 1: AUROC = 0.542, p = 0.560; Purdue 2: AUROC = 0.564, p = 0.423; Purdue 3: AUROC = 0.517, p = 0.828; Purdue 4: AUROC = 0.608, p = 0.083) were not statistically significant.

**Conclusion:**

The DMDA test presented the best performance, with statistical significance and acceptable discrimination, showing greater effectiveness for assessing students’ manual dexterity. Therefore, the implementation of the DMDA test can significantly contribute to the early identification of motor dexterity difficulties in dental students, enabling more effective and targeted interventions.

## Introduction

In Dentistry preclinical training allows competencies and skills to be developed in a controlled and safe environment [1, 2]. These initial trainings are challenging for students who need to put into practice the theoretical content learned for the development of their manual dexterity [3, 4]. Thus, it is necessary that before starting these trainings the level of manual dexterity of each student is evaluated to ensure that those with less dexterity can receive greater support and supervision [5, 6].

Manual dexterity can be conceptualized as the ability to synchronize muscle movement and vision [6, 7], being considered a central component of hand function [8, 9] and fundamental for the dentist [10]. It has been the target of several studies in recent years [1–11], since it is very important for the execution of dental treatment [8, 12].

The most used methods for assessing manual dexterity are not specific to dentistry and, among them, the Purdue Pegboard Test [12–14], O’Connor Finger Dexterity Test [8, 12–14] and the Minnesota Rate of Manipulation [13, 14] stand out. Neves et al. [15] proposed the Dental Manual Dexterity Assessment which is a simple and reproducible method proposed to evaluate the manual dexterity of dental students in the context of Restorative Dentistry.

The manual dexterity assessment methods can assist in the observation of the relationship between motor skills and performance required for preclinical activities in the Dentistry course [4, 16]. However, few studies have used specific tests for Dentistry [16] which makes it difficult to know which is the most effective for preclinical dental training [4, 17].

When preclinical training in restorative dentistry begins, many students lack sufficient manual dexterity and self-confidence to face the initial challenges of practical learning. This can lead to difficulties in performing preclinical procedures [18, 19]. The development of manual dexterity is essential for dental practice. However, if this process is not adequately managed, it can negatively affect the student’s self-confidence and, consequently, their preclinical performance and clinical learning [11, 18–20].

Considering the direct impact of manual dexterity on clinical performance and the quality of dental care, it is crucial to assess students’ level of manual dexterity before the commencement of preclinical training. This will ensure that those with lower dexterity levels receive the necessary support and supervision [5, 6]. Therefore, the present study aims to verify the effectiveness of manual dexterity assessment methods for preclinical dental training, filling a significant gap in the existing literature and providing valuable insights for the improvement of dental education.

## Materials and methods

### Study and sample design

This was an experimental laboratory study with a non-probabilistic sampling design. The response variable was the manual dexterity, measured by the methods O’Connor Manual Dexterity Test—Model 32021; Purdue Pegboard Test—Model 32020A; Dental Manual Dexterity Assessment—DMDA, Class One Cavity Preparation Assessent–COCA, and Class One Composite Resin Restoration Assessment—COCRA. The sample consisted of students enrolled in the last year of the undergraduate course of the São Paulo State University (UNESP), School of Dentistry, Araraquara (N = 95). The students’ recruitment started on July 1^st^, 2021 and ended on May 31^st^, 2022.

The inclusion criteria were students in the last year of the dentistry course, while students from other years did not participate. The tests were carried out at the Pre-Clinical Laboratory of Dentistry and Endodontics at the São Paulo State University (UNESP), School of Dentistry, Araraquara. All sessions were carried out under controlled conditions: artificial lighting, temperature of 22°C (air conditioning) and controlled and standardized noise, without the use of vacuum pumps and compressors. Trained professionals guided the students to carry out the tests individually at their respective workstations, sitting on the dental stool.

This study was approved by the Research Ethics Committee of the School of Dentistry, São Paulo State University (UNESP), Araraquara, Brazil (registration number CAAE: 07990918.0.0000.5416). Written consent was obtained from all participants.

### Preclinical restorative procedures

Before carrying out the manual dexterity tests, the students were asked to perform a Class I cavity preparation on an intact artificial tooth 36 (left lower first molar) and a Class I restoration on an artificial tooth 46 with a factory-standardized Class I cavity preparation (right lower first molar) of a dental mannequin with a phantom head (MOM brand—Dental Manikins Marília).

To carry out the restorative procedures, students were instructed to follow the guidelines of the Dentistry I course of the São Paulo State University (UNESP), School of Dentistry, Araraquara. For the cavity preparation a #1014 round diamond bur was used at low speed. For the restoration, it was used an Almore Suprafill spatula for titanium resin (Duflex) and a #1 double-ended carver (Millennium—Golgran).

### Class One Cavity Preparation Assessent—COCA

The quality of the cavity preparation was evaluated using the Class One Cavity Preparation Assessent—COCA [18], by a properly calibrated researcher (ρ = 0.762), using direct vision. The design, mesiodistal length, buccolingual axis length, depth and roundness of the internal angles of the cavity preparation were evaluated and classified as adequate, partially adequate or inadequate. After that, each item received a score ranging from zero to two points. At the end of the evaluation, the score of all items was added and the cavity preparation received a final score ranging from zero to ten points.

### Class One Composite Resin Restoration Assessment—COCRA

The assessment of the composite resin restoration was performed using the Class One Composite Resin Restoration Assessment—COCRA [21], by a properly calibrated researcher (ρ = 0.762), using direct vision. The presence and evidence of central grooves, the presence and evidence of secondary grooves, the angle of the buccal slopes, the angle of the lingual-palatal slopes, the fabrication of the mesial fossa, the fabrication of the distal fossa, buccal marginal adaptation, lingual-palatal marginal adaptation, mesial marginal adaptation and distal marginal adaptation were evaluated and classified as adequate, partially adequate or inadequate. After that, each item received a score ranging from zero to one point. At the end of the evaluation all items were added, totaling a maximum of ten points.

### O’Connor Manual Dexterity Test (Model 32021)

To perform the O’Connor Manual Dexterity Test, the specific device for the test was used [22]. This device contains in its upper part a shallow concavity that serves as support for the 315 pins to be used in the test. After reading the manufacturer’s instructions, the examiner requested that the evaluated student perform a training prior to the test, placing thirty pins to fill the ten holes of the top line. Then, the test started and the time for its execution was controlled by a timer, observing the seconds needed to fill the device. The time record was divided into 2 parts, with the first part recording the time taken to position the first fifty holes and the second the time for the remaining fifty holes [8, 22].

To calculate the manual dexterity score for this test, it was done the sum of the seconds used to fill the holes in the second half of the device multiplied by 1.1 with the seconds needed to fill the holes in the first half of the device divided by two.

### Purdue Pegboard manual dexterity test (Model 32020A)

For the Purdue Pegboard test a specific device was used which consists of a rectangular plate [23]. In the upper part, this plate has concavities for the storage of the pins, the washers and the collars used in the test and in the middle and lower part two rows parallel to each other, containing 25 holes each. The test includes four tasks: in the first three tasks the participant must insert as many pins as possible into the plate holes in 30 seconds and in the last task in 60 seconds. For each of the tasks, the number of pins inserted represents the score. The Purdue subtest 1 must be performed using the dominant hand, the Purdue subtest 2 the non-dominant hand, the Purdue subtest 3 with both hands, each holding a pin and inserting them into two holes simultaneously, and the Purdue subtest 4 with both hands in an assembly task where the participant needs to build four parts starting with a pin, passing a washer and a collar and ending with an additional washer.

The calculation of the manual dexterity score for the Purdue Pegboard test is performed separately for each test: Purdue subtests 1 and 2—correspond to the number of pins inserted in the test period for each hand; Purdue subtest 3—consists of the total number of pin pairs inserted; Purdue subtest 4—refers to the number of the parts assembled [23].

### Dental Manual Dexterity Assessment—DMDA

The DMDA test was proposed by of Neves et al. [15] and consists of inserting a fine-tipped bur (diamond #3195) into 82 small targets printed and adhered to a styrofoam plate, simulating the entry of the bur into a small carious lesion.

The calculation of the final test score is based on the score attributed to the penetration accuracy of the 82 targets, totaling a maximum of 246 points. Each target evaluated can be scored with 3 points, when penetration occurs fully in its center, 2 points, when it touches its edge occupying more than 50% of it, 1 point, when it touches its edge occupying less than 50% of it and zero for totally off-target penetration [15]. At the end the scores are added up.

The precision of the target penetration was evaluated by a calibrated and blinded researcher (ρ = 0.852), ensuring the reliability of the results.

### Statistical analysis

The tests that assessed the quality of Class I cavity preparation and restoration for composite resin performed (COCA and COCRA) were established as the gold standard. For this, the mean of the scores obtained in the two tests above was calculated, obtaining a single score, and the manual dexterity was dichotomized into satisfactory or unsatisfactory using the value 5.0 as cut-off point (which, in general, is the mean used by dental schools for approvals in the disciplines). Receiver–operating characteristics (ROC) curves were constructed to evaluate the accuracy of each dexterity test for the diagnosis of manual dexterity of dentistry students prior to pre-clinical training. Comparison of the areas under the ROC curves (AUROCs) was performed according to the DeLong et al. [24] and confidence intervals estimation, with a significance level of 5%. All analyzes were performed using MedCalc Statistical version 13.2.0 (MedCalc Software bvba, Ostend, Belgium) software. The criterion adopted for the classification of the discriminatory capacity of the tests was that proposed by Hosmer, Lemeshow [25].

## Results

The majority of the participating students were women (74.7%), with an average age of 21.5 years.

**Fig 1:**
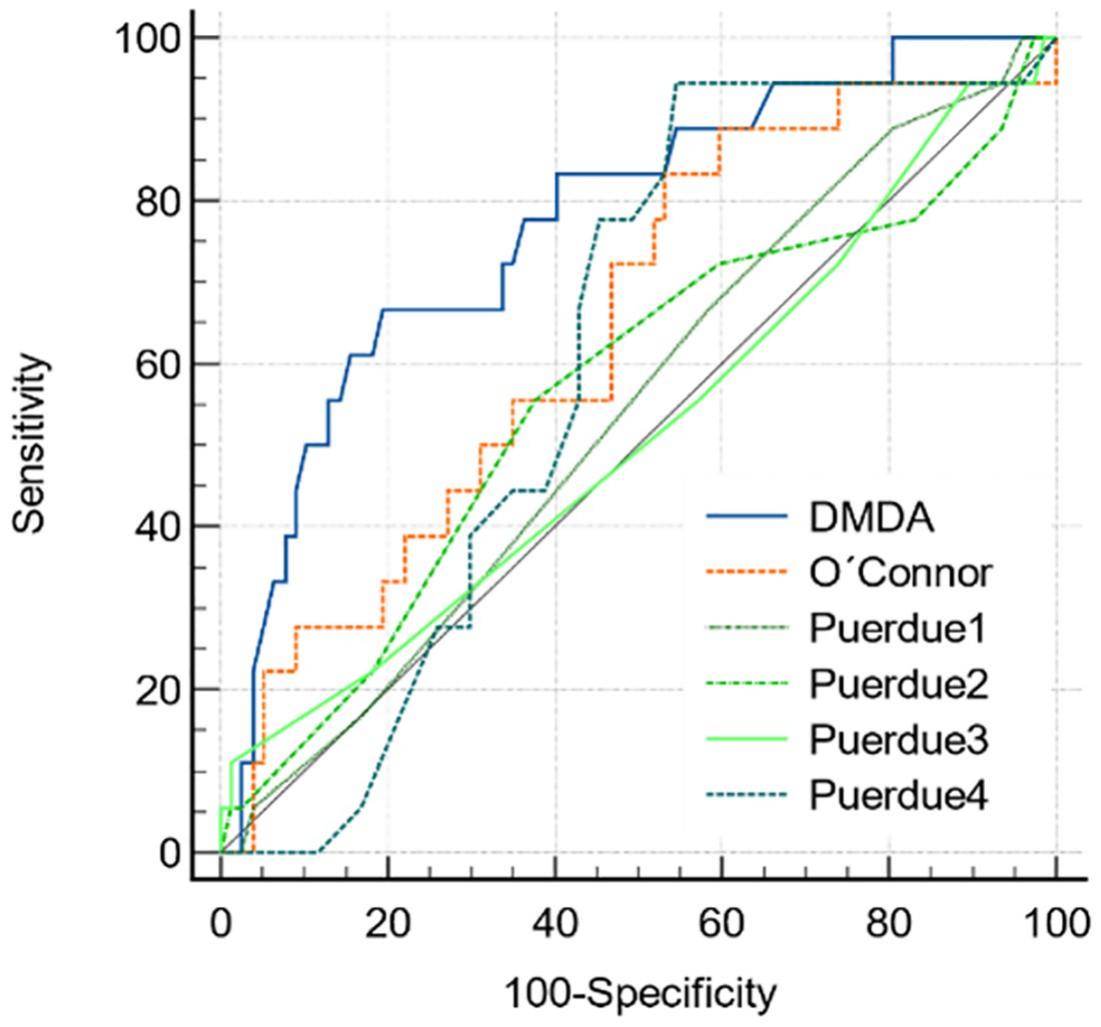
ROC curve for DMDA, O’Connor, Purdue 1, Purdue 2, Purdue 3 and Purdue 4 tests. The mean of COCA and COCRA was considered as the gold standard. **DMDA**: AUROC = 0.775, p<0.001*, IC95% = 0.677–0.854; **O’Connor**: AUROC = 0.644, p = 0.050, IC95% = 0.539–0.739; **Purdue 1:** AUROC = 0.542, p = 0.560, IC95%I = 0.436–0.645; **Purdue 2:** AUROC = 0.564, p = 0.423, IC95% = 0.459–0.666; **Purdue 3:** AUROC = 0.517, p = 0.828, IC95% = 0.412–0.621; **Purdue 4:** AUROC = 0.608, p = 0.083, IC95% = 0.503–0.707.

Fig 1 shows the ROC curve constructed using the DMDA, O’Connor, Purdue 1, Purdue 2, Purdue 3 and Purdue 4 dexterity tests as methods and the mean of COCA and COCRA as gold standard.

The DMDA test showed better performance, with statistical significance (p<0.001) and value obtained at the upper limit of 95%CI, presenting acceptable discrimination (AUROC = 0.775). For all other tests, the area values were not statistically significant, which can be confirmed by CIs that are very close or cross the value of 0.5.

**Fig 2:**
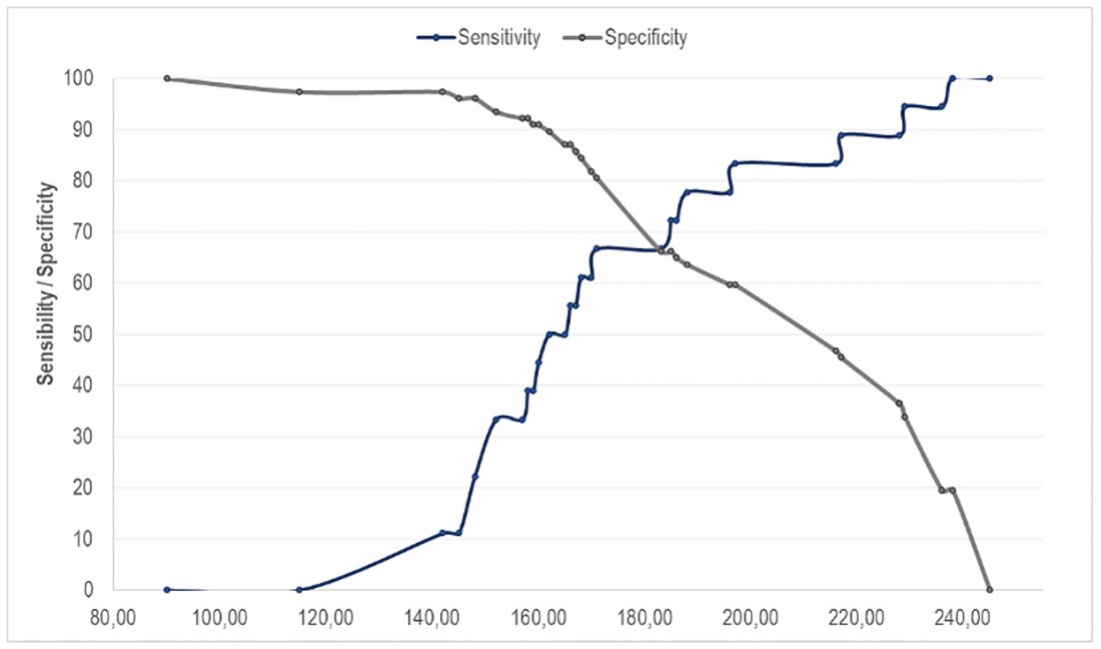
Two Graphic Receiver Operating Characteristic (TGROC) of the sensitivity and specificity values of the DMDA test, according to the cutoff point.

Fig 2 shows the relationship between the sensitivity and specificity values of the DMDA test, according to the cutoff point.

The graph shows that the 183 cutoff point places the sensitivity and specificity balanced at 66.23%. However, it appears that the next point (185) maximizes a little more sensitivity (72.22%) and maintains specificity (66.23%). As sensitivity is more important for this diagnosis (detecting cases of lack of dexterity), this cutoff point was chosen.

## Discussion

Considering that motor skill plays a key role in dental practice [1, 2, 4, 9, 10, 16, 26, 28], the identification of appropriate methods for assessing the manual dexterity of dental students is extremely important [16]. In this context, the present study aimed to verify the effectiveness of three methods of evaluation of manual dexterity (DMDA, O’Connor, Purdue Pegboard) for preclinical training in dentistry.

It was possible to observe that, among the evaluated methods, the DMDA was the one that presented the best performance in the detection of manual dexterity among the evaluated dental students (p<0.001, AUROC = 0.775). Among the three methods tested, DMDA is the only one that was developed specifically to evaluate fine motor skill in preclinical training in Restorative Dentistry, considering the penetration precision of small targets for this [15]. Although it has a quantitative approach related to the sum of the scores assigned to each of the targets penetrated, the analysis of the precision of the penetration of the targets is performed qualitatively, ranging from less accurate, when the bur penetrates completely outside the target, to the most accurate, when the penetration of the bur is centered and inserted fully into the target.

On the other hand, the O’Connor test evaluates dexterity for the rapid manipulation of small objects [8, 12–14] and the Purdue Pegboard the unilateral and bilateral dexterity of the fingers and hand [12–14]. Although the manipulation of small objects and the movement of the fingers and hand occur during dental treatment, the precision of the movement seems to be more important than its speed [8].

This capability likely enhances the DMDA’s ability to predict the presence or absence of manual dexterity before training, allowing educators to tailor the learning process to individual students’ motor skill levels and specific challenges.

Although in the literature there are comparative analyzes between methods of evaluation of dexterity, it is important to note that no research related to the DMDA method was found. Berger et al. [12] compared the O’Connor test and the Purdue Pegboard for work with gloves in a dry and humid environment and observed that the O’Connor test showed better discrimination, especially in a humid environment. The authors justify this difference between the tests by the difference in the diameter of the pins, with those of the O’Connor test having a smaller diameter (1.60 mm) compared to those of the Purdue Pegboard (2.94 mm).

Lugassy et al. [4] used the O’Connor test and the Purdue Pegboard to assess the manual dexterity of dental students and found that the O’Connor test, especially in indirect vision, was more effective in assessing dexterity in preclinical procedures. This is due to the fact that the subtests of Purdue have a very short duration, not reproducing the time spent in dental work, unlike the O’Connor test.

According to Lundergan et al. [8], the assessment methods developed by the Johnson O’Connor Research Foundation have not been completely effective in assessing dexterity in dentistry. They identified a negative relationship between manual dexterity scores using the Johnson O’Connor Tweezer Dexterity Test (a pure speed test) and the quality of restorative procedures.

In the educational environment, the selection of the manual dexterity assessment method should consider, in addition to the effectiveness in its detection and reliability, factors such as cost, time required for its application, practicality, applicability, availability and familiarity [27, 28]. The results of this study indicate that the DMDA test fits these requirements and can be used in preclinical training for the diagnosis of students with greater difficulties in the development of manual dexterity, enabling the implementation of strategies that help them reach the level of skill necessary for the transition to clinical training [15].

The implementation of the DMDA test at the beginning of preclinical training can help identify students with lower levels of manual dexterity. Early intervention strategies, such as specific practice sessions and additional tutoring, can be developed to effectively address these deficiencies. This ensures that students receive the necessary support from the outset of their training, facilitating the development of essential skills.

Based on the results, educators can create personalized training programs that focus on improving specific areas of manual dexterity. This personalized approach not only increases students’ confidence but also enhances their practical performance, leading to better clinical outcomes throughout the course.

Future research should include longitudinal studies to track the long-term effectiveness of the DMDA test in predicting clinical performance. These studies would provide more comprehensive data on the utility and efficacy of this method in dental education, allowing for continuous adjustments and improvements to the method.

The limitation of this study is the non-probabilistic sampling design. However, given the lack of research in this area, the results obtained in this study represent a substantial contribution to the field of teaching in dentistry, particularly with regard to preclinical training. However, it is crucial to emphasize the need to conduct new research to evaluate the effectiveness of this method in various educational contexts.

## Conclusion

The DMDA test presented the best performance, with statistical significance and acceptable discrimination, presenting greater effectiveness for assessing the students’ manual dexterity.

This indicates that the DMDA test can be a valuable tool in preclinical training, helping to identify and address gaps in the manual dexterity of dental students. In this way, it can contribute to improving the preparation of future professionals, addressing current challenges in dental education, and ensuring a higher quality of clinical practice.

## Supporting information

S1 File (PDF)
