# Supplementary material for: Effectiveness of manual dexterity assessment methods for preclinical training in Dentistry
Source: PLoS One. 2024 Dec 4;19(12):e0311973. doi: 10.1371/journal.pone.0311973 (PMC11616867; doi:10.1371/journal.pone.0311973)
Supplement: S1 File — (PDF) [file pone.0311973.s001.pdf]

Supplementary Information

Effectiveness of manual dexterity assessment methods for preclinical training in Dentistry

Luis Eduardo Genaro[1], Tamíris da Costa Neves[2] , Júlia Margato Pazos[2], Lívia Nordi Dovigo[2] e Patrícia Petromilli Nordi Sasso Garcia[2*]

[1] Postgraduate Program in Collective Health in Dentistry, São Paulo State University (UNESP), School of Dentistry, Araçatuba, São Paulo, Brazil.

[2] Department of Social Dentistry, São Paulo State University (UNESP), School of Dentistry, Araraquara, São Paulo, Brazil

*Corresponding author: Patrícia Petromilli Nordi Sasso Garcia

Table S1. Minimal data set of the study findings reported in the article.

Student ID	COCA and COCRA mean	DMDA	O'Connor	Purdue subtest 1	Purdue subtest 2	Purdue subtest 3	Purdue subtest 4
1	5.75	152	334.25	0.50	0.47	0.47	0.63
2	6.00	215	311.25	0.53	0.50	0.50	0.70
3	4.00	188	287.65	0.60	0.50	0.40	0.53
4	5.50	165	305.15	0.53	0.47	0.40	0.68
5	3.50	229	316.65	0.53	0.53	0.37	0.60
6	5.25	173	332.00	0.50	0.43	0.37	0.62
7	4.25	160	321.55	0.50	0.47	0.43	0.68
8	5.25	168	259.55	0.53	0.50	0.47	0.67
9	3.50	171	275.75	0.57	0.53	0.47	0.65
10	3.00	217	295.05	0.50	0.43	0.43	0.47
11	5.00	170	254.00	0.60	0.37	0.43	0.40
12	6.75	191	291.25	0.57	0.47	0.37	0.68
13	6.00	218	290.40	0.47	0.37	0.33	0.47
14	4.25	137	344.85	0.53	0.53	0.37	0.58
16	2.25	142	397.45	0.63	0.50	0.43	0.60
18	6.75	186	284.55	0.60	0.40	0.40	0.73
21	5.50	159	285.20	0.57	0.47	0.43	0.72
23	6.00	221	268.35	0.57	0.50	0.40	0.72
25	6.50	167	304.10	0.53	0.50	0.43	0.68
26	5.00	90	323.05	0.63	0.50	0.40	0.62
27	7.75	170	315.15	0.50	0.47	0.47	0.63
29	3.00	147	325.95	0.53	0.50	0.40	0.65
30	8.75	177	254.05	0.57	0.50	0.40	0.48
31	6.50	179	289.30	0.67	0.47	0.40	0.50

Student ID	COCA and COCRA mean	DMDA	O'Connor	Purdue subtest 1	Purdue subtest 2	Purdue subtest 3	Purdue subtest 4
32	5.50	200	285.90	0.50	0.43	0.33	0.60
33	4.75	152	303.15	0.57	0.50	0.47	0.68
34	6.50	212	459.50	0.47	0.47	0.33	0.27
35	7.50	188	349.90	0.47	0.43	0.37	0.47
36	3.00	148	336.10	0.53	0.47	0.37	0.57
37	7.25	145	289.20	0.60	0.53	0.50	0.70
38	4.25	158	302.85	0.57	0.50	0.47	0.68
40	6.25	229	349.20	0.37	0.33	0.27	0.47
41	8.25	217	297.90	0.57	0.50	0.37	0.60
42	6.75	183	295.10	0.50	0.40	0.37	0.50
43	7.50	212	264.30	0.60	0.53	0.47	0.68
44	6.25	200	282.50	0.53	0.50	0.40	0.63
45	7.25	171	284.65	0.57	0.53	0.47	0.70
46	9.25	229	323.90	0.60	0.50	0.43	0.77
47	3.50	152	400.75	0.50	0.47	0.47	0.63
48	9.75	176	261.90	0.57	0.53	0.50	0.68
49	6.50	162	273.75	0.67	0.63	0.47	0.63
50	4.00	197	303.00	0.53	0.50	0.40	0.68
51	6.00	195	290.95	0.53	0.40	0.33	0.62
52	5.50	202	320.60	0.57	0.53	0.53	0.70
54	5.00	152	253.75	0.53	0.57	0.57	0.53
55	8.00	183	239.90	0.60	0.47	0.43	0.73
56	6.75	181	322.75	0.57	0.50	0.50	0.68
58	2.75	166	232.90	0.57	0.63	0.53	0.70
59	5.75	230	243.95	0.50	0.53	0.43	0.83
60	5.25	181	293.45	0.57	0.43	0.43	0.72
61	6.75	115	342.95	0.60	0.53	0.43	0.68
62	6.75	164	349.95	0.50	0.40	0.37	0.55
63	5.75	173	326.45	0.57	0.53	0.40	0.67
64	6.25	196	310.65	0.50	0.47	0.50	0.60
66	6.75	180	254.80	0.53	0.40	0.47	0.77
68	4.50	185	422.00	0.47	0.37	0.30	0.40
69	6.75	157	246.30	0.53	0.47	0.47	0.67
70	4.75	168	376.75	0.47	0.40	0.23	0.40
71	6.25	174	255.90	0.57	0.50	0.47	0.77
72	4.00	162	292.20	0.53	0.37	0.33	0.55
73	6.25	234	355.60	0.53	0.43	0.37	0.20
74	7.75	232	261.85	0.50	0.43	0.40	0.18
75	5.50	216	282.10	0.53	0.43	0.37	0.20
76	8.00	240	361.30	0.53	0.33	0.37	0.17

Student ID	COCA and COCRA mean	DMDA	O'Connor	Purdue subtest 1	Purdue subtest 2	Purdue subtest 3	Purdue subtest 4
77	6.75	240	295.35	0.67	0.43	0.40	0.17
78	6.50	243	463.20	0.47	0.43	0.33	0.15
79	8.00	233	251.35	0.60	0.47	0.50	0.18
80	6.50	228	283.70	0.53	0.43	0.37	0.17
81	7.25	236	333.85	0.57	0.47	0.33	0.18
82	9.25	230	405.95	0.47	0.43	0.33	0.17
83	8.75	243	372.50	0.47	0.40	0.33	0.17
84	6.00	232	382.00	0.43	0.43	0.43	0.22
85	5.50	210	436.45	0.47	0.37	0.33	0.13
86	8.00	223	306.10	0.57	0.47	0.40	0.25
87	8.00	241	270.85	0.57	0.53	0.47	0.23
88	9.00	228	318.40	0.47	0.43	0.40	0.22
89	6.00	221	319.25	0.40	0.40	0.33	0.18
90	6.25	232	357.25	0.53	0.47	0.37	0.18
91	6.50	240	357.30	0.53	0.43	0.37	0.17
92	6.50	240	307.10	0.53	0.47	0.43	0.20
93	7.25	211	339.35	0.50	0.43	0.33	0.13
94	3.75	238	411.65	0.43	0.40	0.33	0.13
95	7.75	241	279.90	0.57	0.53	0.40	0.22
96	6.25	202	387.15	0.53	0.47	0.33	0.15
97	5.00	243	376.85	0.53	0.50	0.43	0.17
98	7.75	234	297.95	0.53	0.40	0.33	0.22
99	8.00	244	284.95	0.50	0.50	0.40	0.15
100	8.00	236	257.50	0.53	0.43	0.40	0.20
101	6.25	223	258.05	0.60	0.50	0.47	0.25
102	8.50	241	266.00	0.47	0.43	0.37	0.20
103	7.75	239	276.75	0.57	0.50	0.47	0.20
104	9.00	239	276.90	0.63	0.53	0.43	0.22
105	6.50	243	257.50	0.60	0.53	0.43	0.22
106	5.50	236	310.30	0.47	0.43	0.37	0.13
107	8.25	245	303.40	0.53	0.47	0.37	0.22

COCA: Class One Cavity Preparation Assessent; COCRA: Class One Composite Resin Restoration Assessment; O´Connor: O´Connor Manual Dexterity Test (Model 32021); Purdue: Purdue Pegboard Manual Dexterity Test (Model 32020A); DMDA: Dental Manual Dexterity Assessment
